# Do Faculty Members Apply the Standards for Developing Gifted Students at Universities? An Exploratory Study

**DOI:** 10.3390/ejihpe12060043

**Published:** 2022-06-02

**Authors:** Fathi Abunasser, Rommel AlAli

**Affiliations:** 1 Department of Educational Leadership, Faculty of Education, King Faisal University, P.O. Box 400, Al-Ahsa 31982, Saudi Arabia; fabonasser@kfu.edu.sa; 2 The National Research Center for Giftedness and Creativity, King Faisal University, P.O. Box 400, Al-Ahsa 31982, Saudi Arabia

**Keywords:** gifted development standards, gifted, educational programs, gifted development

## Abstract

Many studies indicate the importance of the management and nurturing of giftedness. They also focus on talent development, primarily, where the main objective is enhancing academic abilities. To achieve this goal, it is necessary to explore the reality of faculty members’ application of talent development standards; this is necessary for laying the practical foundations to enhance the academic abilities of talented students according to the standards for verifying quality, and for clarifying the skills and concepts that are taught. The current study was based on the opinions of 122 faculty members from Saudi public universities who had experiences with gifted students, whereby they answered the following question: Do faculty members apply the standards for developing gifted students at universities? The data were collected by developing an instrument. The data were analyzed using descriptive statistics that mostly showed the reality of the application of the selected gifted development standards. The results of the perceptions of the faculty members participating in the study showed differences in the application of the proposed gifted development standards according to their academic rank.

## 1. Introduction

University education institutions are currently facing pressures and challenges represented in rapid growth in the field of knowledge; the great development in communication systems and means; the information and technological revolution; and sudden and rapid changes, such as professions in the labor market, due to the dependence on modern technology and the growing interest in the value of excellence and quality. Therefore, it has become necessary for universities to work on the introduction of these modern systems to ensure survival and continuity in the competition with local, regional, and international universities [1]. Universities are among the most important drivers of change and development in their societies. They play an important role in development at the national level in all fields. Thus, most universities currently operate in a complex, dynamic, and highly competitive global environment. In addition to the presence of those trends related to globalization, there is increasing academic mobility, and an interest in developing academics gifted in many disciplines [1]. Universities’ contributions to development can take various forms, such as developing themselves and the components of their educational and research systems to transform into a knowledgeable society [2].

The academically gifted are considered the most common gifted students in educational systems, and the identification of gifted students depends on the field of giftedness and the stage of gifted development. For example, in some fields of giftedness such as music, art, and mathematics, giftedness is evident early [1]. In this case, ability assessment and gifted development begin according to the methods used. In other fields, such as writing and science, giftedness may appear early, but assessment and gifted development begin in the primary stage [3,4]. Giftedness represents a distinct quality of human energies through which they can achieve progress in society and face the challenges imposed by changing times. Therefore, attention to the gifted becomes a necessity imposed by development, changes, and global conditions, and is a major requirement for any educational system. Therefore, many countries have directed their attention and care toward gifted students. Investing in giftedness is an investment for the future, whereby it achieves the development of productive workers in society [4]. Therefore, societies are keen to identify and care for gifted people by establishing centers, projects, and various institutions, and providing support to detect gifted people, provide them with appropriate programs, and develop their creative thinking skills and abilities [5].

Academically gifted students need an attractive environment, and engaging and challenging learning opportunities to succeed to their fullest potential. Therefore, it is necessary to provide appropriate levels of challenge and enrichment to enable them to exceed their current mastery, to reach a higher cognitive level of thinking and achievement for the success of the academic gifted development model. In addition to the developed distinctive curricula, other aspects of supporting gifted students include teacher instructions, the effective use of time, purposeful assessment, and a change in the prevalent values related to gifted development [6].

### 1.1. Theoretical Framework

Universities are considered one of the most important drivers of change and development in their societies. They play an important role in the development of all fields. Universities also seek to provide care and integrated development for gifted students in various fields, whether inside or outside the classroom, and this care often extends outside the campus walls. The care of gifted students must be provided by providing support for their giftedness, developing creative thinking strategies, and helping them to innovate and invent [7]. 

Because of the challenges and rapid growth in the field of knowledge, the great developments in communication systems, and the information and technological revolution, the role of faculty members is directed and organized to help students acquire and employ knowledge, in addition to developing their abilities, thinking, personality, and giftedness. Universities are responsible for preparing qualified and trained human cadres, which is a vital component of all comprehensive community development processes, and they are one of the most suitable environments for implementing the concept of gifted management [8,9].

Nurturing and developing giftedness in higher education are of great importance, so many studies have discussed this aspect from the perspective of human resource management [10,11]. Globally, there are many attempts in different countries to develop giftedness, and honor programs in American universities are a model for this [11,12]. The word “gifted” refers to academically gifted undergraduate students [13], and it intersects with talented and superior students in most cases. Many European universities have programs for gifted development, which are few compared to American universities, as indicated in the study by [14].

Although universities have many advantages, materials, human capabilities, and infrastructure, they, unfortunately, have not performed their role optimally in developing giftedness and innovation [15]. A study by Al-Ahous showed a deficiency in the care of gifted students in Saudi universities in the organizational aspect or aspects of psychological and scientific care [16]. The importance of gifted development for university students lies in the fact that they are in a stage of mental production; they are the closest group to supporting the wheel of development and innovation, and they have research and scientific skills and the ability to innovate in areas more closely related to their specializations [6]. They also have insight into their strengths and weaknesses and know the fields in which they should excel. Caring for gifted students increases the feeling of loyalty and belonging to their universities.

Many gifted students face many challenges, and there is evidence that failure to adapt to the requirements of the university environment harms the academic performance of gifted students as they move from high school to university life, as shown in the study by Almukhambetova and Hernández-Torrano [17]. Gifted people also face many fears when thinking about their future, and often, problems such as indecision, neurotic perfectionism, and various personal, family, and societal pressures prevent them from expressing their giftedness at university [18].

King Faisal University is considered one of the first Saudi universities to nurture gifted students and develop their skills since the opening of the National Center for Giftedness and Creativity Research, the first research center specialized in giftedness and creativity research, in 2009. The university also initiated the creation of an acceleration system for gifted and talented students in the preparatory year, which is concerned with providing support for gifted students in the subjects of science, mathematics, and English language. The gifted students who pass exams for some courses and classes are exempt. The university has also taken the initiative to develop the skills of gifted students through the Mawhibi Kfu program, which is concerned with enriching giftedness at the university in the categories of creative leadership, cognitive creativity, creative research, and technical creativity [19].

The concept of gifted development is a relatively recent one and was formulated by the study of Axelrod [20], wherein the concept included organizational efforts to attract, develop, and retain gifted individuals. The concept of gifted development is also important in organizational framework and organizational success. Gagné also proposed a concept for gifted development based on the Differentiated Model of Giftedness and Talent (DMGT), which distinguished between giftedness and talent. Giftedness is the natural abilities or aptitudes that characterize the student, and which exist in one or more intellectual, creative, social, cognitive, or physical field. This ranks them in the top 10% of their age peers. Talent is the student’s outstanding performance or competence (knowledge and skills) in one or more fields of human activity that ranks the student among the top 10% of his peers in the same field. The proposed concept of gifted development includes its transformation of outstanding abilities (talents) into distinguished competencies (giftedness) in any professional field [21].

Based on the differentiated model of giftedness, the concept of gifted development for higher education is based on three foundations: giftedness, university infrastructure, and curricula [22]. In addition, Gagné previously defined gifted development as the systematic pursuit of programs and activities by gifted people during a specific period to achieve the desired goal, which is excellence [23]. From the above, it can be noted that the definition includes basic elements: the enrichment training curriculum or program; having a clear, distinct objective; the existence of selective access criteria; systematic and regular practice; the regular and objective assessment of progress; and finally, personal speed and acceleration.

Gifted development includes many components, interests, and programs in order to achieve its development goals. As the gifted development, process consists of three main components: First, gifted development activities that begin when the individual reaches the programs organized for these specialized activities within specific educational coordination, and an investment that is determined by time, and psychological and financial energy. Finally, the progress [24]. Based on the Differentiated Model of Giftedness and Talent, gifted development includes luck or chance; distinctive natural abilities in the mental fields (intellectual, creative, social, and cognitive) and physical fields (movement and muscle control), as well as through environmental stimuli (resources, individuals, and surroundings); internal stimuli such as physical characteristics (health, disability, and physical appearance); mental traits (temperament, personality, and resilience); and the goal of management (awareness, motivation, and will). Then comes the development process, which includes—as we talked about previously—activities, investment, and progress. Finally, we obtain academic competencies (technology, science and technology, art, social services, and commercial operations), games, sports, and athletics [23]. In addition, gifted development includes the personal development of individuals, including their abilities, skills, competencies, and motivations. Gifted development focuses on identifying and implementing individual organizational capabilities, interests, and goals. It should also be closely linked with human resource development activities such as attracting and retaining giftedness for organizational success [25]. Conger focused on four components of gifted development: individual-skill development, social-development interventions, work, and strategic-learning initiatives [26].

Academic giftedness has many definitions according to the school of thought. Gagné has adopted two definitions of academic gifted development [27]. The first definition states that academic gifted development is a long-term structured program of longitudinal learning activities in the academic curriculum with a continuous challenge to achieve distinctive high-level goals. The second definition states that academic gifted development is the systematic pursuit of long-term personal goals of excellence by the academically gifted student within the program. Studies indicate that to achieve or form best practices that promote the best academic achievement, it is necessary to identify the basic characteristics that constitute these practices. It was noted that there is a large group of gifted programs (acceleration, summer camps, weekend activities, etc.) and a great diversity of practices for these programs, but there is little homogeneity between them. In contrast, the practices of gifted development in the fields of art, music, and sports have great and wide homogeneity and convergence [28]. Therefore, best practices have been identified to implement academic gifted development programs as follows: curriculum enrichment, the complete and permanent assembly of abilities, customized acceleration, early interventions, and achieving personal excellence. Benjamin Bloom pointed to the importance of external variables such as motivation and encouragement to follow the gifted development process, and internal variables such as desire and strong interest in practice and training, commitment to the gifted field, and the ability to learn quickly and well [4,24].

Gifted development faces many challenges during the implementation of these programs. These challenges have been the field of study of many interested people and researchers, including the weak link between the student’s personality and their interests, the lack of diversity between students’ abilities, and impeding the development of the relationship between teachers and students [25]. In addition, there are challenges in the measurement and evaluation processes; these require gifted development professionals, in general, to adhere to specialized standards and theories to the fullest, and to apply strategies and evaluation tools in an elaborate and professional manner, to obtain the desired results from the gifted development process.

Achieving gifted development requires the availability of standards for gifted development, as countries pay great attention to nurturing and developing gifted and talented students. There is no doubt that there is an urgent need for specific criteria for gifted development programs. To design these programs, a set of criteria must be considered. Standards are a set of indicators and services that are achieved and available in programs related to gifted development. The importance of standards in education generally highlights the fact that they emphasize the quality of education and describe what learning and teaching should be, to improve outcomes. They provide the education system with a basis for evaluation and provide equal opportunities for education for all gifted people [29,30]. The National Association for Gifted Children (NAGC) and the Council for Exceptional Children (CEC) are among the first educational institutions to focus on preparing gifted programs and include seven criteria: learner growth and individual differences in learning; learning environments; curriculum content knowledge; assessment; teaching planning and strategies; professional learning and ethical practices; and collaboration [31,32,33,34].

The National Center for Measurement and Evaluation (Qiyas) put a set of standards related to the gifted field, including teaching methods and strategies for gifted and talented students, information technologies, technology in education, and scientific methods, organized to evaluate gifted students and the programs offered to them [29,35]. As for the Saudi Mawhiba criteria, it was based on six criteria for gifted programs according to the American Association for Gifted Children NAGC, and the criteria included learning and development, assessment, curriculum planning, teaching, learning environments, programming, and finally, professional development [36].

The study by VanTassel-Baska and Hubbard reviewed eight gifted programs using program criteria (NAGC) in various states. The results showed that the regions achieved just over 50% of the indicators for the three criteria. For the other three criteria, the gifted programs in the region met only 31% to 37% of the indicators. The fields of greatest need include further program development, the development of planning-tools for curriculum implementation, monitoring the effectiveness of program implementation, establishing routine use of content acceleration in all subjects, and developing counseling support for gifted learners [37].

Several studies have discussed the topic of gifted development. Paula Olszewski-Kubilius demonstrates the basic principles underpinning the new gifted development framework, whereby general ability is a basis for developing more specific and relevant capabilities for different gifted fields, recognizing the gifted development framework which states that different academic fields have unique pathways [4]. The gifted development framework emphasizes the deliberate nurturing of psychosocial skills that support high achievement, rather than leaving it to chance [38]. The study by Gagné also analyzed Academic Gifted Development (ATD) from two distinct perspectives: theoretical and practical. Each of these perspectives can be summarized in the following questions. What are the personal and contextual causal influences that contribute most to the emergence of excellence in subjects? What educational resources will further transform outstanding abilities into academic excellence? These two questions will guide the contents of this chapter [27]. A study by Prickel et al. aimed to describe and explain achievement and its development in different fields of gifted development, and it showed the possibility of building gifted development models for the fields of mathematics, music, and visual arts [39]. Meanwhile, the purpose of a study by Luis et al. was to investigate the role of psychosocial support training for gifted development in sports. Four elite Brazilian athletes, two men and two women, were interviewed. The participants evaluated psychosocial support training as essential for developing their giftedness. They highlighted the following dimensions of training: biofeedback, mindfulness techniques, and feelings of knowing what to do under stress [40]. The results of this study indicated that the factors associated with psychosocial support training, such as psychological strength, mastery orientation style, and tactical discipline, may contribute to the outstanding performance of players. When psychosocial support training is applied to elite players, it may improve psychosocial performance and development. The theoretical and practical implications of this study are discussed, taking into consideration the development of giftedness in various fields. The study by Ziegler et al. clarified the role of mentors or supervisors for gifted students at different levels and developmental fields. Fields differ in terms of start, peak, and end time. Therefore, mentoring at the beginning of the giftedness path may be directed at various gifted students according to the later stages of gifted development, as the trainees acquire the required skills and knowledge, and mentors focus more on the modeling and training of psychosocial skills and internal knowledge [41].

From the above, the need to determine the degree of the practice of standards for gifted development in university education emerges from the development of an integrated system to care for and build giftedness and creative national competencies that contribute to the growth of the Kingdom, and shift it towards a knowledgeable society; this requires concerted efforts to establish advanced programs for gifted students, as universities have an important role in this context.

### 1.2. Study Problem

Universities strive to achieve better academic services for gifted students and move away from traditional lecture-oriented education and the curriculum book, which is based on the lower levels of thinking. They work to increase the development of creative thinking using modern teaching methods, such as investigation and discovery—which is reflected in their programs and activities—to be a guides that work on developing creativity. The orientation of gifted youth can be viewed from a developmental perspective, and in comparison, with the role of teaching and guidance towards acquiring psychological and social skills and providing internal knowledge. The current study seeks to reveal the approved standards for the activities and events of gifted programs and the standards for the development of the academically gifted, by surveying the opinions of faculty members at King Faisal University and challenging the reality of their practice. Specifically, the current study attempts to answer the following main question: What is the degree of application of gifted development standards for faculty members at King Faisal University, from their point of view?

### 1.3. Study Questions

The following sub-questions are derived from the main question:

1. What is the degree of application of gifted development standards for faculty members at Saudi Universities, from their point of view, in the following dimensions: learner development and individual learning methods; learning environments; curricular content; assessment; instructional planning and strategies; professional learning and ethical practice; and cooperation?
2. Are there any significant differences in the degree of application of gifted development standards by faculty members at Saudi Universities, from their point of view, due to gender, academic rank, teaching experience, and college?

### 1.4. Significance of the Study

The importance of this study stems from the importance of defining a frame of reference for activities and events, and programs of the gifted, by revealing the degree to which faculty members apply the standards for gifted development to keep pace with contemporary international and global trends; it is concerned with educating and nurturing the gifted and talented, and the urgent need to localize gifted programs with strategic plans through the national standards for their care. These standards include learner development and individual learning methods, learning environments, curricular content, assessment, instructional planning and strategies, professional learning and ethical practice, and cooperation.

### 1.5. Definition of Terms

#### Gifted Development

Gifted development is defined as planning, selecting, implementing, and applying development strategies for several gifted students [18]. It is defined procedurally for this study and includes the academic development of students, as well as their abilities, skills, competencies, and motives.

## 2. Methodology 

### 2.1. Approach

The study used the descriptive analytical approach because of its connection with the study and realism in dealing with the research problem. The results and data on the faculty’s application of university talent-development standards are presented in a quantity that is easily understood.

### 2.2. Participants

The population of this study consisted of all faculty members and lecturers at Saudi Universities during the second semester of the academic year 2021/2022, these universities were distributed over five regions: the north, south, east, west, and middle of the Kingdom of Saudi Arabia. The sample of this study constituted a random selection of faculty members from all colleges at Saudi Universities, and consisted of 122faculty members and lecturers who cooperated with gifted students. Only faculty members participating in gifted student programs at these universities were targeted, and the sample of the study varied according to the following variables: gender, academic rank, experience, and college type. Table 1, below, shows the distribution of the study sample according to its variables.

Table 1 shows a fair distribution in the college-type variable (54 for humanities colleges, 68 for scientific colleges), while the levels of other variables reflect the status quo in universities and the natural ratios of this distribution, and represent similar and acceptable ratios.

### 2.3. Procedures

After developing the initial perception of the study, a review of the scientific literature on the subject of the study was collected; then, the development of the study instrument and its arbitration were performed. In addition, to ensure the honesty, validity, and stability of the study instrument, so that it became applicable, the approval of the Committee on the Ethics of Scientific Research at the University and approvals for the application were obtained. The team started collecting data. Originally, five universities from the study population were selected on purpose to re-express discontent from different geographical regions, and provided with educational materials and an instrument link distributed through WhatsApp. The instrument was published in the form of a Google model, so that the responses could be collected in an excel file. Colleagues from the five universities helped with the distribution process. The WhatsApp message included instructions and reassurances for the respondents from the faculty members. They were assured that their answer would be in accordance with the teaching practices of gifted students, and they were informed of the approval of the Scientific Research Ethics Committee at King Faisal University. In addition, the form included the same instructions for confirmation. The instrument was re-applied at universities where participants had a poor response, with researchers personally reaching out to faculty members to encourage responses, and with the help of their colleagues from those universities. The research team was keen to represent the study sample. 

### 2.4. Instrument

An instrument was developed after reviewing the literature and previous studies. The objective was defined and the dimensions of the instrument were determined; then, the items of instrument were developed. Some of the criteria and items from studies contained in the theoretical framework were used to develop the study instrument [4,14,31,33,37]. The instrument was used to measure the degree of application of gifted development standards by faculty members at Saudi Universities, from their point of view [39]. It consisted of seven dimensions, namely, learner development and individual learning methods (7 items), learning environments (7 items), curricular content (7 items), assessment (7 items), educational planning and strategies (7 items), professional learning and ethical practice (7 items), and cooperation (7 items). The final copy of the instrument consisted of 49 items (Appendix B).

The validity and reliability of the instrument were verified. Nine experts from Saudi University examined the instrument items. Based on their opinions, the researchers modified and reformulated some instrument items, and also omitted some items. To ensure validity and reliability, the instrument was piloted with 23 faculty members, and the responses and feedback obtained were used in modifying the final instrument. The data were analyzed using SPSS version 26. To find the validity of the instruments, the discriminant coefficient (corrected item-total correlation) was determined using SPSS. The items that had a discriminant coefficient of less than 0.20 were omitted. Sample responses were used in the calculation of matrix correlation coefficients between the sub-scales. The total score, as shown in Appendix A, represents the matrix correlation coefficients between the mean of the sub-scales (learner development and individual learning methods; learning environments; curricular content; assessment; educational planning and strategies; professional learning and ethical practice; and cooperation) and the mean of the total score (overall average). It also shows that the values of correlation coefficients are relatively high (0.913–0.397); this indicates that all sub-scales were involved in the measurement of a single concept of gifted development, and emphasizes the correlation of sub-scores with the total score.

To verify the factorial construct validity of the instrument, an exploratory factor analysis (EFA) was performed using the principal component method. Oblique rotation was carried out using the Varimax method to obtain the factors, by selecting the most loading items for each factor. The factor analysis resulted in 7 loading factors on its 49 items, and the value of its total variance was 74.762%; this percentage is considered high. 

The factorial construct validity of the instrument was also verified, and the loading items assumed for each dimension with the dimension that measured these items. The sample responses on the scale items were subjected to confirmatory factor analysis (CFA) with the maximum likelihood method using the Amos program, and the analysis confirmed that the scale included 7 dimensions, and each dimension contained 7 items. Further, the loading values ranged from 0.469–0.944, which indicated the factorial validity of the scale, as shown in Appendix C.

Cronbach Alpha: Cronbach’s Alpha Based on Standardized Items for the whole dimension was 0.936. This value indicates that the instrument has a good degree of reliability, and ranked the study-tool items using the following equation: (the highest value of the alternative-minimum alternative)/number of levels; (5 − 1)/5 = 0.80. The Likert scale has five options or values: 1, 2, 3, 4, and 5. The goal of this classification is to classify responses. Therefore, the levels as follows: very low (1.0–1.8), low (1.81–2.6), medium (2.61–3.41), high (3.42–4.22), and very high (4.23–5.0).

### 2.5. Data Analysis

To answer the study questions, we employed the means; standard deviation to determine the rank and the degree to which faculty members apply the gifted development standards; and a t-test one-way analysis of variance to determine differences, in addition to using Tukey’s test as a post hoc test. 

## 3. Results

This section includes a presentation of the results of this study. To answer the first question, the instrument dimensions about the degree of application of gifted development standards were analyzed. The means, standard deviation, rank, and degree to which faculty members apply the gifted development standards were extracted. Table 2, below, shows the means, standard deviation, rank, and degree to which faculty members apply the gifted development standards on the whole scale.

Table 2 shows that the item scores in the professional learning and ethical practice dimension ranked first and had a mean of 4.46 and standard deviation of 0.58 This dimension includes treating students in a fair and appropriate manner, instilling confidence in them, instilling values, building a leadership personality, and motivating them for scientific research. On the other hand, it stimulates their freedom of expression. The lowest dimension is “Educational Planning and Strategies” with a mean of 3.66. This dimension refers to clear strategies and programs for the development of various talents, such as planning to enhance and develop students’ talents and cooperate with colleagues, using pre-determined strategies for different learning situations, and long-term planning when designing a lesson to develop students’ skills and talents.

In general, the mean of the professional learning and ethical practice dimension indicated a very high degree of practice. Meanwhile, the item scores in dimensions of assessment; learning environments; curricular content; learner development and individual learning methods; cooperation; and educational planning and strategies had means of 4.15, 4.06, 3.96, 3.91, 3.8, and 3.66, respectively, and standard deviations of 0.616, 0.59, 0.60, 0.73, 0.67 and 0.79, respectively. In general, the means of these dimensions indicated a high degree of practice.

To answer the second question, a t-test and one-way analysis of variance were used. Table 3, below, shows the results of the t-test for the degree to which faculty members apply the gifted development standards in the dimensions of the scale based on gender and faculty.

Table 3 shows a value of t = 0.001 for the whole dimension, indicating that there were no statistically significant differences between the means. In other word, there were no statistically significant differences between the responses of the sample to the degree to which faculty members apply the gifted development standards, according to gender. The sub-domains did not include any significant differences either. Additionally, regarding the college variable, the value of t = 2.495 for the whole dimension indicates that there were statistically significant differences between the means, where the significance level was less than (0.01). In other words, there are statistically significant differences in the sample responses to the degree of application of the criteria for gifted development in the dimensions of the overall scale. Regarding sub-domains, “cooperation” showed significant differences in the humanities and sciences faculties, and yet, it did not affect the overall dimensions. 

Table 4 below shows the results of the one-way analysis of variance for the degree to which faculty members apply the gifted development standards in the dimensions of the scale, due to the academic rank and teaching experience.

The results in Table 4 show that there are statistically significant differences at the level of significance (0.01) in the responses of the study sample members about the application of the standards of development for gifted people in universities, based on academic rank in the overall field and in the following sub-fields: learner development and individual learning methods; learning environments; curricular content; educational planning and strategies; and cooperation. Additionally, the table shows that there were no differences in the following sub-fields: assessment, and professional learning and ethical practice. To determine the sources and trends of the differences, Tukey’s test was used for the post-comparisons was used.

From Table 5, it appears that there are statistically significant differences in the degree of application of the dimensions of the talent development standards scale, based on teaching experience. Moreover, these differences are in favor of experienced faculty members and in favor of the faculty members with the most teaching experience, i.e., “More than 10 years”.

Table 6 shows that there were statistically significant differences in all dimensions of the degree of application of the gifted development standards, based on academic rank. These differences are in favor of faculty members with the rank of professor among various other academic ranks. 

## 4. Discussion

The study aimed to determine the degree of application of gifted development standards for faculty members at King Faisal University, from their point of view. This was discussed by answering some questions. Below is a discussion of the results related to these questions.

### 4.1. Results Related to the First Question

Table 2 shows that all fields of the instrument obtained a high degree of agreement; the mean values ranged between 3.6663–4.4637, and the field of professional learning and ethical practice ranked first, with a very high degree. This indicates the interest of the study sample members in the ethics of professional learning and ethical practice among university students, in addition to the keenness of the faculty members to instill confidence among students. In addition, faculty members showed a keenness to instill confidence in students, respect students and their opinions, allow freedom of opinion for students, and work to develop students’ research skills according to ethical rules and local and international standards. This is in line with the basic principles on which Saudi universities are based, which consider ethical practices as a mainstay that the university leadership is keen to promote among faculty members, as well as to students and university employees. In addition, these professional practices in student education are in line with basic principles that are compatible with the teaching profession in universities, and with the recommendations of international organizations—especially UNESCO and the Arab Education Office for the Gulf Cooperation Council countries—to form an integrated system that frames the educational practices of university faculty members.

Universities can invest in gifted development by focusing on the fields described in this study, considering the specific environment of each university and the level of services it provides to gifted and talented students; this certainly contributes to the development of the gifted. It also contributes to the specific standards at each university for services to gifted students, which often follow the policy of caring for the gifted at these universities and provide an umbrella of care based on global standards in the field.

Meanwhile, the mean values of other fields related to the learning environment; assessment; learner development and individual learning methods; cooperation; and planning and educational strategies indicated, to a high degree, the interest of faculty members in applying the standards of gifted development at the university, from their point of view. It reflects their keenness on the standards of gifted development and achieving the university’s mission to support and motivate the academically gifted at the university, as well as diversifying the practices that push the framework of gifted development and taking care of them in an institutional way. This may be due to the university administration’s continuous encouragement of its employees towards creativity in various educational practices, and the provision of moral and financial incentives and scientific prizes for distinguished students in this aspect. Additionally, it may be due to parental roles in the faculty’s dealings with gifted and talented students [42].

It may also be due to the subjective feeling of the faculty members towards the students and their desire to distinguish them, whereby they seek to localize the culture of giftedness, creativity, innovation, and excellence in them.

Although the field of educational planning and strategies ranked last, with a mean of 3.6663, it also achieved a high degree. This may explain the presence of many faculty members with the rank of assistant professor, and those with relatively little experience—where their experiences are in applying the vocabulary of this field in a small way—in terms of the university having clear plans and programs to develop different gifts, and in terms of using pre-defined educational strategies and having long-term plans when designing lessons to develop students’ skills. This may be because the pattern of application of these practices was not conducted in the required manner, due to the many academic and administrative burdens on faculty members, particularly since this study was conducted under exceptional circumstances—especially considering the coronavirus pandemic, which witnessed a shift towards electronic education in whole or in part. Therefore, it increased the teaching and academic burden on faculty members.

### 4.2. Results Related to the Second Question

The results of the t-test showed that there were statistically significant differences among the responses of the sample according to gender, in favor of female faculty members; this has also been evidenced by other studies [18]. This result may be because females are more serious and more emotional in dealing with gifted students, and they are the closest to abiding by regulations and instructions in a patriarchal culture. Seriousness is a prominent feature of female faculty members in Arab universities and is an opportunity for them to prove themselves in societies classified as masculine; this is an incentive to outperform their male colleagues in many areas, including the archaeological programs of talented students, according to other Arab studies [29]. They are also keen on motivating students, providing an encouraging environment based on individual planning, paying more attention to educational content, diversifying assessment strategies, and cooperation. In addition, they are more keen to apply the ethics of scientific research than males. These practices provide women with a greater degree of distinction and a greater ability to compete under the full control of male faculty members over various aspects of academic life at the university, at the level of leadership positions and at the level of committees and programs, as previous studies have suggested [30]. Moreover, these differences may be somewhat small, with the university applying quality procedures in most academic and educational processes, especially with the universities keeping pace with the transformation processes taking place in the Kingdom of Saudi Arabia in terms of attention to education and its quality, stimulating and developing giftedness, creativity, and innovation. This can be achieved through the implementation of high-quality services [30].

Table 3 shows that there were no statistically significant differences due to the type of college (humanities, scientific). This result is consistent with the university’s orientations in generalizing all educational and academic practices to the various colleges, regardless of their type. It is also consistent with the availability of all programs, training courses and activities in the context of the development of talented people, creativity, and innovation, for various faculty members at humanitarian and scientific colleges, where many studies show the importance of training in the context of the development of faculty capabilities [40]. Therefore, the response of the faculty members came in a similar framework, whether in the scientific or humanities colleges. We should, therefore, benefit from the etiquette of cognitive competence research and the education of talented people when considering such aspects and trying to deal with them [43]. The presence of statistically significant differences between faculty members in scientific and humanities faculties in the field of cooperation with talented students tends in favor of scientific faculties; moreover, it justifies the nature of educational activities that are often practical and include greater rapprochement and cooperation than other educational activities that take the individual approach in the faculties of humanities. This may also be due to the keenness of faculty members in scientific faculties to cooperate with students in laboratories, in the interest of their safety. Table 4 and Table 5 show that there were statistically significant differences in the response of faculty members based on teaching experience in favor of faculty members who have more than ten years of expertise. This result comes in a natural context, where extensive and long experience in academic university work gives individuals many positive practices in the field of gifted development and achieving its standards, especially when these experiences are linked to practical aspects or practices motivated by university leadership, both at the level of the financial incentives provided by the university. This aspect is very important for universities that aspire to develop giftedness and work to develop their experiences in investing in giftedness, and creating an environment and structure to salute this field or moral awards. Experience has its place and importance in the learning process in general, has its role in outperforming and distinguishing students from their peers, and is positively correlated with academic achievement gains for students throughout a faculty member’s career. This makes faculty members with longer experience true practitioners of academically gifted development processes and events, and their experiences are rooted in the increase in the frequent application of these practices, and through training programs, activities, and events with which the university enriches its employees during their academic career; consequently, they are more aware of the requirements and needs of their students’ skills and abilities. As for the less experienced faculty members, they are on the road and have not reached the same experiences despite the availability of training programs for them; however, the actual practices are still not equal to those with lots of experience. This justifies the differences that increase according to field experience when there is intense competition between faculty members in academic departments, and each category is trying to prove itself against the other. With the increase in experience, the role of the creative faculty member deepens in developing creativity in their students. They present science and knowledge in different and modern ways, create a stimulating climate for intellectual creativity, encourage students to self-evaluate, provide students with the opportunity to express themselves, and are keen to provide them with scientific research skills. This result is due to the fact that more experienced faculty members are more familiar with recent intellectual developments, in addition to their participation in scientific conferences and symposia compared to less experienced faculty members, which justifies these results.

For universities, the programs for nurturing gifted and talented students are concerned with providing an appropriate educational environment for the development of individual and gifted abilities; they can invest in the expertise of the faculty members who are deep in their fields to maximize this idea, by training their inexperienced colleagues and transferring experiences to other administrators who are interested in nurturing the talented.

The results also showed that there were statistically significant differences in the response of faculty members based on academic rank, in favor of Assistant Professor, as shown in Table 4 and Table 6. This may be because assistant professors at the university comprise the vast majority and are the most interested in achieving the standards of academic and educational processes in general, and standards related to gifted development in particular. This is because they want to achieve best practices and satisfy subordinate academic leaders so that they can compete with those who are higher than them at the academic level (associate professor and professor) where competition is different from faith; this corresponds to the results of many studies [29]. They also seek to own and adhere to the strategies of planning-cooperation and evaluation in a uniform and disciplined manner, through the application of the standards for talent development in various fields. They do this to achieve job stability, taking into account the general focus of Saudi universities towards attracting talented students, making their jobs more vulnerable than those with higher academic ranks who have broader experience and whose jobs are more functionally stable. This often occurs in universities where most faculty members’ nationalities are not of the country in which they work. 

From the above, Saudi universities, like some international universities, can move forward in gifted development according to specific standards, and move towards the future with new scientific fields. These standards begin with constructing identification (for those previously identified). To apply international codified scales, a scale codified in the Arab environment (Aurora), which was codified by King Faisal University in cooperation with Yale University, led to the employment of various talent fields in universities according to programs that are also subject to international standards. This procedure enhances the employment of international standards in gifted development, and this experience can be transferred to many universities around the world. The previous results may refer to perceptions of gifted development frameworks in universities so the that scientific and academic promotion of giftedness will be more effective when applying for scientific and enrichment programs. Universities can also set their standards in line with the local environment to achieve better results and excellent care, and many studies have called for this trend to be adopted [37]. These results can be circulated to many Asian and African universities; this is especially the case for universities in the Arabian Gulf region and the Middle East because of the similarity of the environment in which they are taught, and can be utilized in many other countries. The performance of low-grade faculty members can also be improved through training, professional standards for gifted programmers, and their comprehensive application. In addition, university leadership must provide an environment that is conducive to teamwork and innovation to improve the organizational commitment of faculty members toward gifted students [44].

Universities have become increasingly important in terms of entrepreneurship and innovation, activating scientific research centers, building partnerships with the private sector, and providing consultancy and training. One of the concepts that has certainly changed is the university’s role in the innovation system, and the university has been seen as a source of inventions, patents, licenses, and pop-up companies. Intellectual property accounts for only 2% of the total flow of knowledge from the university sector, making it necessary for universities to review gifted development standards and ensure that they are applied, to achieve the objectives of these universities and the regional and global community. Universities should take into consideration the experience of faculty members, their gender, and their academic ranks in their legislation; these factors play a prominent role when implementing any legislation or formulating any new policies, especially those related to education, learning, science, and scientific research.

## 5. Conclusions

Going back to the beginning, do faculty members apply standards for developing gifted students in universities? The results of this study showed that universities differ in many aspects, both in terms of the availability of standards and policies for gifted students, as well as in the degree of application of these standards, if they exist. It is mostly individual faculty members who follow and apply the standards, which calls for a holistic view of the issue. This requires the presence of international institutions that frame these standards (standards for gifted development); it obliges universities to use them according to university classification standards, and requires the presence of mandatory programs. The Academy of Universities promotes the practice of faculty members following these standards. The current study is limited to the perceptions of faculty members and lecturers from Saudi public universities who cooperated with gifted students. Only five Universities were selected from the five regions in the Kingdom of Saudi Arabia.

## 6. Recommendations and Future Directions

Considering the findings of the study, it recommends intensifying targeted training programs to develop the skills of faculty members in relation to the development of gifted students. In addition, it recommends framing gifted development programs and strategies at the university level, generalizing it to colleges and departments, and motivating faculty members to work with them. It also recommends providing institutions that frame these standards. The study attempted to highlight the concept of gifted development in the university environment. It also showed the foundations that frame this process, and this stimulates scientific research in the field of giftedness and the development of creativity at the level of higher education institutions. This study can serve as the beginning of a series of studies that discuss gifted development strategies. It is also possible to test other variables in the university environment that may affect gifted development. 

## Figures and Tables

**Table 1:** The distribution of the study sample.

		Frequency	Percent	Valid Percent	Cumulative Percent
Gender	Male	82	67.2	67.2	67.2
	Female	40	32.8	32.8	100.0
	Total	122	100.0	100.0	
Rank	Lecturer	21	17.2	17.2	17.2
	Assistant Professor	57	46.7	46.7	63.9
	Associate Professor	19	15.6	15.6	79.5
	Prof	25	20.5	20.5	100.0
	Total	122	100.0	100.0	
Experience	Less than 5 years	25	20.5	20.5	20.5
	From 5 to 10 years	22	18.0	18.0	38.5
	More than 10 years	75	61.5	61.5	100.0
	Total	122	100.0	100.0	
Colleges	Humanities Faculties	54	44.3	44.3	44.3
	Scientific Faculties	68	55.7	55.7	100.0
	Total	122	100.0	100.0	

**Table 2:** The means, standard deviation, rank, and degree to which faculty members apply the gifted development standards on the whole scale.

Rank	Dimension	N	Mean	Std. Deviation	Degree of Applicability
1	Professional Learning and Ethical Practice	122	4.4637	0.58523	Very high
2	Assessment	122	4.1511	0.61693	High
3	Learning Environments	122	4.0597	0.59509	High
4	Curricular Content	122	3.9696	0.60169	High
5	Learner Development and Individual Learning Methods	122	3.9110	0.73954	High
6	Cooperation	122	3.8454	0.67995	High
7	Educational Planning and Strategies	122	3.6663	0.79605	High
	Overall average	122	4.0095	0.53237	High

**Table 3:** Results of *t*-test for differences between means according to gender and faculty.

Variables and Dimensions			No.	Mean	Std. Deviation	T-Value	Sig.
Gender	Learner Development and Individual Learning Methods	Male	82	3.9373	0.72569	0.314	0.576
		Female	40	3.8571	0.77372		
	Learning Environments	Male	82	4.0209	0.60071	0.700	0.405
		Female	40	4.1393	0.58275		
	Curricular Content	Male	82	3.9233	0.59822	0.693	0.407
		Female	40	4.0643	0.60518		
	Assessment	Male	82	4.1969	0.60469	0.314	0.576
		Female	40	4.0571	0.63871		
	Educational Planning and Strategies	Male	82	3.6429	0.83518	2.584	0.111
		Female	40	3.7143	0.71685		
	Professional Learning and Ethical Practice	Male	82	4.4094	0.64857	9.397	0.003
		Female	40	4.5750	0.41238		
	Cooperation	Male	82	3.7944	0.69592	0.230	0.632
		Female	40	3.9500	0.64178		
	Overall Average	Male	82	3.9893	0.54645	0.001	0.977
		Female	40	4.0510	0.50648		
**Faculty**	Learner Development and Individual Learning Methods	Humanities	54	4.0794	0.71707	2.279	0.024
		Scientific	68	3.7773	0.73484		
	Learning Environments	Humanities	54	4.2513	0.61300	1.128	0.002
		Scientific	68	3.9076	0.53780		
	Curricular Content	Humanities	54	3.9894	0.67967	1.327	0.753
		Scientific	68	3.9538	0.53648		
	Assessment	Humanities	54	4.2672	0.57482	1.179	0.061
		Scientific	68	4.0588	0.63756		
	Educational Planning and Strategies	Humanities	54	3.8466	0.88797	5.922	0.025
		Scientific	68	3.5231	0.68827		
	Professional Learning and Ethical Practice	Humanities	54	4.5344	0.50503	1.191	0.224
		Scientific	68	4.4076	0.63996		
	Cooperation	Humanities	54	4.0767	0.68692	3.500	0.001
		Scientific	68	3.6618	0.61996		
	Overall Average	Humanities	54	4.1493	0.56914	2.495	0.009
		Scientific	68	3.8986	0.47683		

**Table 4:** Results of analysis of variance of differences between the means of responses of the sample.

Variance Source			Sum of Squares	df	Mean Square	F	Sig.
Academic Rank	Learner Development and Individual Learning Methods	Between Groups	15.623	3	5.208	12.155	0.000
		Within Groups	50.554	118	0.428		
		Total	66.177	121			
	Learning Environments	Between Groups	4.137	3	1.379	4.203	0.007
		Within Groups	38.714	118	0.328		
		Total	42.851	121			
	Curricular Content	Between Groups	5.123	3	1.708	5.209	0.002
		Within Groups	38.683	118	0.328		
		Total	43.805	121			
	Assessment	Between Groups	2.274	3	0.758	2.043	0.112
		Within Groups	43.779	118	0.371		
		Total	46.053	121			
	Educational Planning and Strategies	Between Groups	14.570	3	4.857	9.227	0.000
		Within Groups	62.108	118	0.526		
		Total	76.678	121			
	Professional Learning and Ethical Practice	Between Groups	1.149	3	0.383	1.122	0.343
		Within Groups	40.292	118	0.341		
		Total	41.441	121			
	Cooperation	Between Groups	10.745	3	3.582	9.351	0.000
		Within Groups	45.197	118	0.383		
		Total	55.942	121			
	Whole Dimensions	Between Groups	5.811	3	1.937	8.025	0.000
		Within Groups	28.482	118	0.241		
		Total	34.293	121	5.208		
Teaching Experience	Learner Development and Individual Learning Methods	Between Groups	27.118	2	13.559	41.309	0.000
		Within Groups	39.059	119	0.328		
		Total	66.177	121			
	Learning Environments	Between Groups	18.524	2	9.262	45.307	0.000
		Within Groups	24.327	119	0.204		
		Total	42.851	121			
	Curricular Content	Between Groups	12.172	2	6.086	22.895	0.000
		Within Groups	31.633	119	0.266		
		Total	43.805	121			
	Assessment	Between Groups	11.935	2	5.967	20.813	0.000
		Within Groups	34.118	119	0.287		
		Total	46.053	121			
	Educational Planning and Strategies	Between Groups	27.045	2	13.523	32.422	0.000
		Within Groups	49.633	119	0.417		
		Total	76.678	121			
	Professional Learning and Ethical Practice	Between Groups	4.784	2	2.392	7.765	0.001
		Within Groups	36.657	119	0.308		
		Total	41.441	121			
	Cooperation	Between Groups	16.012	2	8.006	23.859	0.000
		Within Groups	39.931	119	0.336		
		Total	55.942	121			
	Whole Dimensions	Between Groups	14.836	2	7.418	45.370	0.000
		Within Groups	19.457	119	0.164		
		Total	34.293	121	13.559		

**Table 5:** Results of Tukey’s test for differences between the length of experience of faculty members in relation to application of the gifted development standards.

Mean	(I) Experience	(J) Experience	Mean Difference (I-J)	Std. Error	Sig.
3.3486	Less than 5 years	From 5 to 10 years	−0.63473 *	0.11820	0.000
		More than 10 years	−0.88898 *	0.09338	0.000
3.9833	From 5 to 10 years	Less than 5 years	0.63473 *	0.11820	0.000
		More than 10 years	−0.25425 *	0.09804	0.029
4.2376	More than 10 years	Less than 5 years	0.88898 *	0.09338	0.000
		From 5 to 10 years	0.25425 *	0.09804	0.029

* Significance level (0.01).

**Table 6:** Results of Tukey’s test for differences between the periods of academic rank of faculty members for application of the gifted development standards.

Mean	(I) Rank	(J) Rank	Mean Difference (I-J)	Std. Error	Sig.
3.9786	Lecturer	Assistant Professor	0.17196	0.12541	0.520
		Associate Professor	−0.34898	0.15556	0.118
		Prof	−0.27771	0.14543	0.230
3.8067	Assistant Professor	Lecturer	−0.17196	0.12541	0.520
		Associate Professor	−0.52095 *	0.13015	0.001
		Prof	−0.44967 *	0.11785	0.001
4.3276	Associate Professor	Lecturer	0.34898	0.15556	0.118
		Assistant Professor	0.52095 *	0.13015	0.001
		Prof	0.07128	0.14953	0.964
4.2563	Prof	Lecturer	0.27771	0.14543	0.230
		Assistant Professor	0.44967 *	0.11785	0.001
		Associate Professor	−0.07128	0.14953	0.964

* Significance level (0.01).

## Data Availability

The authors declare that all other data supporting the findings of this study are available within the article and its supplementary information files. Informed consent was obtained from all individual participants included in the study.

## Appendix A

**Table A1:** Correlation matrix of the scale.

		AvLD	AvLE	AvMC	AvA	AvPS	AvPE	AvC	AvTOT
AvLD	Pearson Correlation	1	0.735 *	0.820 *	0.637 *	0.639 *	0.470 *	0.539 *	0.862 *
	Sig. (2-tailed)		0.000	0.000	0.000	0.000	0.000	0.000	0.000
AvLE	Pearson Correlation	0.735 *	1	0.769 *	0.669 *	0.612 *	0.597 *	0.614 *	0.877 *
	Sig. (2-tailed)	0.000		0.000	0.000	0.000	0.000	0.000	0.000
AvMC	Pearson Correlation	0.820 *	0.769 *	1	0.716 *	0.739 *	0.544 *	0.571 *	0.913 *
	Sig. (2-tailed)	0.000	0.000		0.000	0.000	0.000	0.000	0.000
AvA	Pearson Correlation	0.637 *	0.669 *	0.716 *	1	0.491 *	0.560 *	0.397 *	0.780 *
	Sig. (2-tailed)	0.000	0.000	0.000		0.000	0.000	0.000	0.000
AvPS	Pearson Correlation	0.639 *	0.612 *	0.739 *	0.491 *	1	0.300 *	0.775 *	0.827 *
	Sig. (2-tailed)	0.000	0.000	0.000	0.000		0.001	0.000	0.000
AvPE	Pearson Correlation	0.470 *	0.597 *	0.544 *	0.560 *	0.300 *	1	0.232 *	0.633 *
	Sig. (2-tailed)	0.000	0.000	0.000	0.000	0.001		0.010	0.000
AvC	Pearson Correlation	0.539 *	0.614 *	0.571 *	0.397 *	0.775 *	0.232 *	1	0.747 *
	Sig. (2-tailed)	0.000	0.000	0.000	0.000	0.000	0.010		0.000
AvTOT	Pearson Correlation	0.862 *	0.877 *	0.913 *	0.780 *	0.827 *	0.633 *	0.747 *	1
	Sig. (2-tailed)	0.000	0.000	0.000	0.000	0.000	0.000	0.000	

* Significance level (0.01).

## Appendix B

**Table A2:** Dimensions and items of the scale.

First: Learner Development and Individual Learning Styles.						
N	Items	Applicable				
		Very High	High	Moderate	Low	Very Low
1.	I provide an educational learning environment capable of developing creative thinking skills					
2.	I provide all the resources and tools needed for effective learning					
3.	I activate the learning environment to ask my questions as clearly as possible					
4.	I spread an atmosphere of fun among the students during the lecture					
5.	I provide the emotional and social climate for the students to facilitate the management of the lecture					
6.	I enhance research skills, critical thinking skills, dialogue and interaction skills, and self-learning among gifted students in a learning environment					
7.	I promote new ways of learning in focus groups and diverse classes from multiple angles and open to reality					
Second: Learning Environments						
1.	I provide an educational learning environment capable of developing creative thinking skills					
2.	I provide all the resources and tools needed for effective learning					
3.	I activate the learning environment to ask my questions as clearly as possible					
4.	I spread an atmosphere of fun among the students during the lecture					
5.	I provide the emotional and social climate for the gifted students to facilitate the management of the lecture					
6.	I enhance research skills, critical thinking skills, dialogue and interaction skills, and self-learning among gifted students in a learning environment					
7.	I promote new ways of learning in focus groups and diverse classes from multiple angles and open to reality					
Third: Methodological Content						
1.	I am working on that the content of the courses includes skills that develop students’ talents					
2.	I use methods based on curriculum maps to develop gifted					
3.	I am working on updating the courses periodically and enriching them with new and creative content					
4.	I link educational content to students’ needs and their external environment					
5.	I present ideas using different techniques to attract gifted students’ interest					
6.	I address students’ interests in an original and creative way					
7.	I provide flexible approaches to content, teaching, and output.					
Fourth: Evaluation						
1.	I seek fairness and transparency when evaluating students’ skills to develop talents					
2.	I continuously and appropriately assess the student’s progress.					
3.	I use a variety of assessment methods appropriate to the educational content.					
4.	I work to stimulate creative thinking in the student through appropriate assessment methods					
5.	I use more than one method in evaluating one skill					
6.	I make sure that the evaluation is inclusive of all elements of the educational situation					
7.	I use various assessment strategies to develop students’ educational talents					
Fifth: Instructional Planning and Strategies						
1.	The university has clear strategies and programs to develop different talents					
2.	I plan to enhance and develop students’ talents through teaching strategies					
3.	I collaborate with colleagues in the specialization to model student learning and provide what develops their skills					
4.	I am working on the existence of a prior plan for teaching the course in a way that enhances gifted development					
5.	I use predefined strategies for different educational situations					
6.	I create the necessary creative teaching requirements by fully designing the educational situation, and then practicing mentally performing this position.					
7.	I plan for the long term when designing the lesson to develop students’ skills and talents					
Sixth Dimension: Professional Learning and Ethical Practice						
1.	I treat students fairly and appropriately					
2.	I strive to instill confidence in the students					
3.	I use the scientific material to instill values and build character					
4.	I am developing the professional side of my gifted students with the latest research and readings					
5.	I respect the students’ answers and accept them no matter how correct they are, and I do not make fun of them					
6.	I allow students to express their opinion and express what is inside them					
7.	I show a high level of morals, because I am an example to students and an example to the university.					
Seventh Dimension: Cooperation						
1.	I cooperate with the university administration in providing learning opportunities to develop talents					
2.	I distribute the tasks to the learning groups appropriately for the levels of the students					
3.	I think cooperative learning is beneficial					
4.	I encourage students to carry out joint research to develop in them a spirit of cooperation					
5.	I cooperate with my colleagues in developing strategic plans to develop students’ talents					
6.	I form a research team of students to study the phenomena of society in my specialty					
7.	I allocate a specific time to respond to students’ inquiries through communication sites and office hours					

## Appendix C

**Table A3:** The results of the confirmatory factor analysis of the scale.

Item	1	2	3	4	5	6	7
LD1	0.889						
LD2	0.861						
LD3	0.855						
LD4	0.763						
LD5	0.750						
LD6	0.639						
LD7	0.557						
LE1		0.494					
LE2		0.469					
LE3		0.942					
LE4		0.901					
LE5		0.895					
LE6		0.892					
LE7		0.777					
MC1			0.668				
MC2			0.659				
MC3			0.549				
MC4			0.824				
MC5			0.821				
MC6			0.810				
MC7			0.799				
A1				0.723			
A2				0.676			
A3				0.673			
A4				0.639			
A5				0.636			
A6				0.587			
A7				0.560			
PS1					0.878		
PS2					0.820		
PS3					0.662		
PS4					0.655		
PS5					0.534		
PS6					0.532		
PS7					0.776		
PE1						0.755	
PE2						0.693	
PE3						0.496	
PE4						0.858	
PE5						0.696	
PE6						0.457	
PE7						0.508	
C1							0.652
C2							0.937
C3							0.718
C4							0.673
C5							0.660
C6							0.712
C7							0.551
